# The Role of Wildfire, Prescribed Fire, and Mountain Pine Beetle Infestations on the Population Dynamics of Black-Backed Woodpeckers in the Black Hills, South Dakota

**DOI:** 10.1371/journal.pone.0094700

**Published:** 2014-04-15

**Authors:** Christopher T. Rota, Joshua J. Millspaugh, Mark A. Rumble, Chad P. Lehman, Dylan C. Kesler

**Affiliations:** 1 Department of Fisheries and Wildlife Sciences, University of Missouri, Columbia, Missouri, United States of America; 2 Forest and Grassland Research Laboratory, United States Forest Service Rocky Mountain Research Station, Rapid City, South Dakota, United States of America; 3 Custer State Park, South Dakota Department of Game, Fish, and Parks, Custer, South Dakota, United States of America; Université de Sherbrooke, Canada

## Abstract

Wildfire and mountain pine beetle infestations are naturally occurring disturbances in western North American forests. Black-backed woodpeckers (*Picoides arcticus*) are emblematic of the role these disturbances play in creating wildlife habitat, since they are strongly associated with recently-killed forests. However, management practices aimed at reducing the economic impact of natural disturbances can result in habitat loss for this species. Although black-backed woodpeckers occupy habitats created by wildfire, prescribed fire, and mountain pine beetle infestations, the relative value of these habitats remains unknown. We studied habitat-specific adult and juvenile survival probabilities and reproductive rates between April 2008 and August 2012 in the Black Hills, South Dakota. We estimated habitat-specific adult and juvenile survival probability with Bayesian multi-state models and habitat-specific reproductive success with Bayesian nest survival models. We calculated asymptotic population growth rates from estimated demographic rates with matrix projection models. Adult and juvenile survival and nest success were highest in habitat created by summer wildfire, intermediate in MPB infestations, and lowest in habitat created by fall prescribed fire. Mean posterior distributions of population growth rates indicated growing populations in habitat created by summer wildfire and declining populations in fall prescribed fire and mountain pine beetle infestations. Our finding that population growth rates were positive only in habitat created by summer wildfire underscores the need to maintain early post-wildfire habitat across the landscape. The lower growth rates in fall prescribed fire and MPB infestations may be attributed to differences in predator communities and food resources relative to summer wildfire.

## Introduction

Western North American forests are shaped by natural disturbances. From small-scale canopy gaps to stand-replacing fires, disturbances are an important source of heterogeneity and species diversity [1]. Wildfires and mountain pine beetle (*Dentroctonus ponderosae*, hereafter MPB) infestations are disturbances of particular interest to managers because of their widespread occurrence and economic impacts. Wildfires burned an average of 2.7 million ha annually between 2001 and 2011 [2]. Many species benefit from wildfires, from xylophagous insects that reproduce in dead and dying trees [3] to various ungulates that benefit from improved forage production [4], [5]. Widespread MPB infestations occur irregularly in western forests, though eruptions impact millions of hectares and last for several years [6], [7]. Benefits of MPB infestations include a superabundant food resource (beetle larvae) that is exploited by many species [8], [9] and wildlife habitat in the form of standing dead trees [7].

Black-backed woodpeckers (*Picoides arcticus*) are emblematic of the role these natural disturbances play in creating wildlife habitat. Black-backed woodpeckers are associated with habitat created by wildfire [10]–[14], prescribed fire [15], and MPB infestations [16]–[18]. Although black-backed woodpeckers are known to occupy undisturbed forests [19]–[21], densities in such habitat is low relative to recently burned forests [11], [20], and woodpeckers may avoid undisturbed forest altogether if recently burned forests are in close proximity [22]. Despite the importance of recently disturbed forests to black-backed woodpeckers, wildfire and MPB infestations reduce timber value and have historically been considered undesirable. Considerable effort is thus put into preventing or mitigating the effects of these disturbances through fire suppression, post-fire salvage logging, and sanitation logging, which may contribute to habitat loss for black-backed woodpeckers [23], [24]. As a result, black-backed woodpeckers are considered a sensitive species in Region 2 of the U.S. Forest Service, a species of greatest conservation concern by the State of South Dakota [25], and have recently been petitioned for listing under the Endangered Species Act [26].

Identifying effective conservation strategies requires understanding the relative value of wildfire, prescribed fire, and MPB infestations to black-backed woodpeckers. However, considerable uncertainty exists regarding the relative value of these disturbances. Many authors consider black-backed woodpeckers a fire-dependent species [12]–[14], [22], [27], [28] that rely on moderate or high severity burns [29], [30]. Despite potential benefits to some wildlife species, forest managers often focus on reducing the incidence of high severity wildfires. Prescribed fire is one tool used to meet this objective [31], but prescribed fire can differ from wildfire in important ways. For example, prescribed fires often burn at low severity, while wildfires often burn at mixed or high severity. Additionally, forests are typically treated with prescribed fire during spring or fall, when fires are easier to control [32], while wildfires often burn during summer months [33]. Such differences in timing can impact post-fire arthropod communities [34], [35], potentially affecting food resources for black-backed woodpeckers. Therefore, while habitat created by prescribed fire may appear similar to habitat created by wildfire, the value of prescribed fire to black-backed woodpeckers remains unknown.

Although typically associated with post-fire habitat, black-backed woodpeckers are also attracted to MPB infestations. Black-backed woodpeckers use MPB infestations in lodgepole pine (*Pinus contorta*) forests in the Cascade Mountains of Oregon [16] and ponderosa pine (*Pinus ponderosa*) forests in the Black Hills of South Dakota [17], [18]. However, use of MPB infestations is not uniform across their range. For example, black-backed woodpeckers were rarely detected in MPB infestations in lodgepole pine forests in the northern Rocky Mountains [36] and in MPB infested lodgepole pine/Douglas fir (*Pseudotsuga menziesii*) forests in British Columbia [9]. Such apparent discrepancies cause uncertainty regarding the value of MPB infestations in various forest types to black-backed woodpeckers.

We evaluated demographic rates of black-backed woodpeckers in habitats created by summer wildfire, fall prescribed fire, and MPB infestations in the Black Hills, South Dakota. We first estimated adult and juvenile survival and reproductive rates of black-backed woodpeckers occupying each of these disturbance types. We then derived habitat-specific population growth rates as a function of demographic parameters. By evaluating habitat-specific population growth rates, we clarify the relative value of each of these disturbances for black-backed woodpeckers in the Black Hills.

## Materials and Methods

### Study Sites

This study occurred in the Black Hills, South Dakota at study sites representing habitat created by wildfire, prescribed fire, and MPB infestations (Table 1). Study sites were selected by first identifying forest patches that were burned by wildfire, burned by prescribed fire, or infested with MPBs. Potential study sites were then searched for signs of woodpeckers, and those study sites where woodpeckers were found were included in the study. Site selection was thus opportunistic because forest patches needed to be both disturbed and occupied by black-backed woodpeckers. Because of the opportunistic nature of site selection, the eastern-most study sites were burned by wildfire and the western-most study sites were infested with MPBs (Table 1). We do not believe such placement affected our results because the Black Hills National Forest is a maximum of 65 miles wide, and we are not aware of any east-west gradients that would influence black-backed woodpecker population dynamics. All wildfire sites burned during June or July (hereafter we use wildfire and summer wildfire synonymously; see Table 1 for the year each wildfire burned) and all prescribed fire sites burned during September or October (hereafter we use prescribed fire and fall prescribed fire synonymously; see Table 1 for the year each prescribed fire burned). All study sites were predominately monotypic stands of ponderosa pine, with occasional quaking aspen (*Populus tremuloides*), paper birch (*Betula papyrifera*), and white spruce (*Picea glauca*) [37]. All prescribed fire and wildfire study sites contained trees that burned at low, moderate, and high severity, although the proportion of trees burned at each severity category varied by study site. All MPB study sites contained trees that had been infested <1 year, 1–2 years, and >2 years. All study sites also contained live trees unaffected by wildfire or MPB infestations. Field work began in April 2008 and continued year-round through August 2011. Additional field work at prescribed fire study sites occurred from May through August 2012.

**Table 1:** Study sites used to evaluate Demography of black-backed woodpeckers in the Black Hills, South Dakota, USA.

Site	Habitat	Coordinates	Size (Ha)^a^	Month/year disturbed^b^	Years included in study
Ricco	Wildfire	44°13′N, 103°25′W	1,602	July 2005	2008, 2010
Box Elder	Wildfire	44°9′N, 103°24′W	129	July 2007	2008, 2009
4-Mile	Wildfire	43°41′N, 103°26′W	955	June 2007	2008–2011
Bullock	Rx fire	44°0′N, 103°30′W	486	Sept. 2008	2010–2012
Bitter	Rx fire	43°58′N, 103°26′W	304	Oct. 2010	2012
Headquarters West	Rx fire	43°34′N, 103°30′W	255	Sept. 2009	2011
American Elk	Rx fire	43°61′N, 103°49′W	1376	Oct. 2010	2012
Norbeck	MPB	43°50′N, 103°30′W	>213^c^	1998	2008
Bear Mountain	MPB	43°51′N, 103°45′W	>48^c^	Before 1995	2008–2011
East Slate Creek	MPB	43°58′N, 103°44′W	>1,303^d^	Before 1995	2008–2011
Deerfield Lake	MPB	44°00′N, 103°49′W	>169^c^	Before 1995	2008
Medicine Mountain	MPB	43°52′N, 103°42′W	>1,748^d^	Before 1995	2009–2011

a Size of MPB infestations calculated from FHP Aerial Detection Surveys, available at <http://www.fs.usda.gov/detail/r2/forest-grasslandhealth/?cid=fsbdev3_041629> (accessed Feb. 13, 2013). This is an estimate of the minimum total area impacted by MPBs in each study site in a given year.

b The first year MPB infestations were detected in FHP Aerial Detection Surveys. Note there is no aerial detection data prior to 1995.

c Calculated from 2008 FHP Aerial Detection Survey.

d Calculated from 2010 FHP Aerial Detection Survey.

### Capture and Radio-telemetry

We used very high frequency radio-transmitters (Advanced Telemetry Systems, Isanti, Minnesota, USA; Lotek Wireless Inc., Newmarket, Ontario, Canada) to collect survival data from adult and juvenile black-backed woodpeckers. We initially targeted adult woodpeckers for capture by searching study sites for signs of woodpeckers. We then used mist nets, hoop nets, and netguns [38] to capture adult woodpeckers. Once captured, we weighed all adults and attached a 3.0–3.3 g transmitter [39]. Adult black-backed woodpeckers weighed an average of 75 g (SD = 5 g), so transmitters weighed <5% of an average adult bird’s mass [40]. We also marked each bird with a unique combination of colored leg bands and a uniquely numbered US Fish and Wildlife Service (USFWS) aluminum leg band. As radio-transmitter batteries failed, we attempted to recapture marked individuals and replace transmitters. We supplemented recaptured birds with unmarked birds that were captured opportunistically. Adult woodpeckers with active transmitters were relocated at least once per month, though most woodpeckers were relocated more frequently (mean number of telemetry locations per month = 6).

We captured black-backed woodpecker nestlings at the nest cavity with a ‘chick-snagging’ device and by accessing the nest with a hole-saw [41]. We constructed chick-snagging devices by looping fishing line through 1.27 cm plastic tubing. At approximately 3-days post-hatching, we captured nestlings with the chick-snagging device by entangling them in the looped fishing line. All nestlings captured with the chick-snagging device were fitted with a unique combination of colored leg bands and a uniquely numbered USFWS aluminum leg band but were not fitted with radio-transmitters. We only used the chick-snagging device during the 2009 breeding season. We captured nestlings during all other years by accessing the cavity with a 7.62 cm hole-saw approximately 3 days prior to fledging. All nestlings captured with the hole-saw method were weighed and fitted with a unique combination of colored leg bands and a uniquely numbered USFWS aluminum leg band. After capture with the hole saw, nestlings were placed back in the cavity and the access hole was plugged. During the 2010 breeding season, all nestlings (*n* = 25) were fitted with a 2.2 g transmitter [39]. During the 2011 breeding season, one randomly selected nestling from each nest was fitted with a 2.2 g transmitter until all available transmitters were used (*n* = 6). Nestlings weighed an average of 57 g (SD = 10 g) and transmitters weighed <5% of an average nestling’s mass [40]. Fledglings with active transmitters were relocated as soon as possible after emerging from the nest and every-other week thereafter. Fledglings without transmitters were relocated by observing radio-marked parents provisioning individually-marked young.

### Reproductive Success and Number of Young Fledged

We located nests by systematically searching study areas, by following birds to their nests, and opportunistically while collecting field data. We visited nests every 3–4 days during the 2009–2011 breeding seasons until either the nest failed or fledged young. Because of logistic constraints, nest visits were less regular during the 2008 and 2012 field seasons and the mean interval between nest visits was 4 days. We examined nest contents with a camera attached to a telescoping pole [42], [43]. We assumed the number of young fledged was the number of nestlings present in the nest during the last visit prior to fledging.

### Ethics Statement

The majority of field work occurred on public land (Black Hills National Forest, Custer State Park, and Wind Cave National Park). We obtained permission to access Wind Cave National Park from the US National Park Service, permit number WICA-2010-SCI-0011. Two authors (MAR and CPL) were employees of the US Forest Service and Custer State Park, respectively. We therefore did not require specific permission to access the Black Hills National Forest or Custer State Park. We avoided private lands when possible, and obtained permission from landowners when necessary to access private land (e.g., when woodpeckers were located on private land).

All birds were individually marked and fitted with transmitters as quickly as possible to minimize stress. All research protocols were approved by the University of Missouri Animal Care and Use Committee, protocol numbers 4439 and 7205. Black-backed woodpeckers are protected under the Migratory Bird Act. Permission to capture and band black-backed woodpeckers was thus obtained from the US Department of the Interior, Federal Bird Banding Permit number 23574 and Federal Fish and Wildlife Permit number MB161645-0; and from the State of South Dakota Department of Game, Fish, and Parks, Free Scientific Collector’s Permit License Number 10.

### Estimating Adult and Juvenile Survival

We estimated adult and juvenile survival probabilities with Bayesian multi-state mark recapture models [44]. We considered a 3-state model where woodpeckers were classified as ‘detected alive’ (state 1), ‘detected dead’ (state 2), or ‘undetected’ (state 3). We assumed the following state-transition matrix:
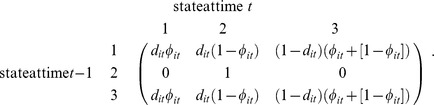
where woodpeckers transition from the state along the row to the state along the column with the associated cell probability. Detection probability, denoted *d_it_*, is the probability woodpecker *i* is detected by the end of time step *t*. Survival probability, denoted φ*_it_*, is the probability woodpecker *i* is alive at the end of time step *t*. We thus modeled a woodpecker’s state at time *t* as a multinomial random variable:
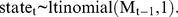
where **M** *_t_*_−1_ is the row of the state transition matrix associated with a woodpecker’s state at time *t*–1.

We modeled adult and juvenile detection and survival probability during each time step as a function of covariates. We assumed a 1-month time step for adult detection and survival probability and a 2-week time step for juvenile detection and survival probability. We modeled adult and juvenile detection probability as a function of whether woodpecker *i* had an active transmitter during time step *t*. Juveniles that were color-banded but did not receive a transmitter were coded as having an inactive transmitter. We modeled monthly adult survival probability as a function of sex, season (breeding, April – September; non-breeding, October – March), the habitat occupied at the end of each time step (wildfire, prescribed fire, and MPB infestation), and number of years post-fire. We modeled bi-weekly juvenile survival probability as a function of habitat, the number of years post-fire, and the number of two-week time intervals since fledging. Details of covariate models and prior distributions are described in Appendix S1. Juvenile detection histories began the last full two-week interval they remained in the nest and lasted through the end of March the following year. Thus, the first transition included the period when juveniles fledged from the nest. Note that woodpeckers originally captured as juveniles were assumed to survive as adults the 1^st^ April post-fledging, which corresponds with the beginning of the breeding season.

Woodpeckers inhabiting established study sites were assigned the habitat category associated with that study site (e.g., woodpeckers at the 4-Mile study site were assigned to wildfire habitat; Table 1), even if woodpeckers were occasionally located in adjacent undisturbed forest (Fig. S1, Fig. S2). Habitat categories thus reflect the type of disturbed forest each woodpecker was primarily associated with, even if they occasionally occupied other forest types during a single time step. Such a coarse categorization of habitat association is appropriate given the coarse scale at which we modeled adult (1 month) and juvenile (2 weeks) survival probability. Note that woodpeckers occasionally dispersed from established study sites, sometimes moving from one disturbance type to another (e.g., moving from a wildfire to a prescribed fire). When such dispersal occurred, we modeled survival probability as a function of the habitat occupied at the end of the time step. We only observed woodpeckers dispersing to forest disturbed by wildfire, prescribed fire, or MPB infestations.

### Estimating Nest Success and the Number of Young Fledged

We modeled daily nest survival probabilities with a Bayesian nest survival model [45]. We modeled daily survival probability as a function of habitat and time since fire (Appendix S1). Detection histories for all nests started the first day a nest was found. Detection histories for successful nests continued until the last date the nest was observed active [46] and detection histories for unsuccessful nests ended the first date nest failure was observed. We treated the days between the last observation of an active nest and the first observation of a failed nest as missing data and imputed the response variable.

We modeled the number of young fledged from each successful nest with a zero-truncated Poisson model. We modeled the number of young fledged per successful nest as a function of habitat (Appendix S1).

### Estimating Habitat-specific Population Growth Rates and Sensitivity Analysis

We calculated habitat-specific annual population growth rates with a 2-stage female-based pre-breeding projection matrix [47]:
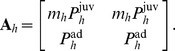
where *m_h_* is habitat specific fecundity (calculated as a function of nest success and the number of young fledged, Appendix S1), 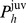 is the habitat-specific probability a juvenile will survive to the adult stage, and 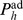 is habitat-specific annual adult female survival probability (Appendix S1). We used life stage simulation analysis (LSA) to evaluate sensitivity of population growth rates to variation in component demographic rates [48] (Appendix S1).

## Results

### Adult and Juvenile Survival

We censored detection histories of adult birds with inadequate data or whose mortality was possibly a result of capture. In total, we censored complete detection histories for 7 adults and censored mortality events for 4 adults that were recovered dead the first observation after a recapture. We estimated survival probabilities from detection histories of 140 adult woodpeckers, which consisted of 369, 406, and 164 cumulative months at risk of dying in habitat created by wildfire, MPB infestations, and prescribed fire, respectively. The average period between first and last detection of individual adults was 8 months (range = 1 to 40 months).

**Figure 1:**
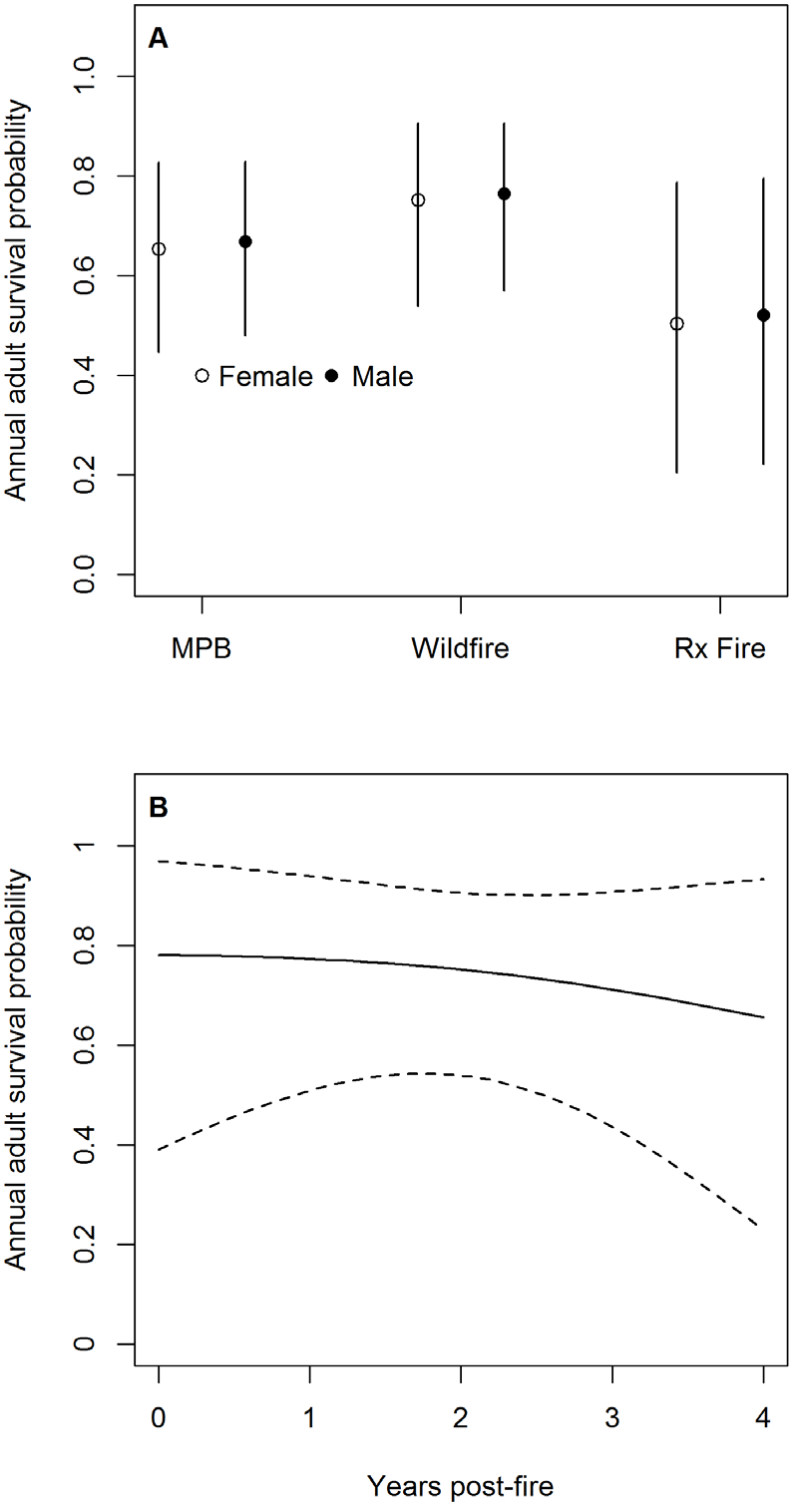
Estimated annual adult survival probabilities of black-backed woodpeckers in the Black Hills, South Dakota . (a) Mean posterior distribution and 95% CI of adult male and female black-backed woodpecker annual survival probabilities in habitat created by wildfire, prescribed (Rx) fire, and mountain pine beetle (MPB) infestations in the Black Hills, SD. Plots of annual survival probabilities assume habitat created by wildfire and prescribed fire are both 2 years post-fire. (b) Mean posterior distribution and 95% CI of annual survival probability as a function of time since wildfire. This figure assumes an adult female in habitat created by wildfire, but the trend is similar in habitat created by prescribed fire.

Adult survival probability was greatest in habitat created by wildfire, intermediate in habitat created by MPB infestations, and lowest in habitat created by prescribed fire (Table S1). For example, mean annual adult female survival probability was 0.75 (95% CI = [0.54, 0.91]) in 2-year post-wildfire habitat, 0.65 (95% CI = [0.45, 0.83]) in MPB infestations, and 0.50 (95% CI = [0.20, 0.79]) in 2-year post-prescribed fire habitat (Fig. 1a). Mean annual adult survival probability was nearly identical between sexes and declined slightly as time since fire increased (Fig. 1b). Finally, mean monthly survival probability was slightly greater during the breeding season relative to the non-breeding season. For example, mean monthly adult female survival probability in habitat created by MPB infestations was 0.97 (95% CI = [0.94, 0.99]) during the breeding season and 0.96 (95% CI = [0.92, 0.98]) during the non-breeding season.

We censored the detection history for 1 juvenile that died before fledging. After censoring, we estimated juvenile survival probabilities from detection histories of 72 woodpeckers, consisting of 119, 139, and 44 cumulative time steps (2-week intervals) at risk of dying in habitat created by wildfire, MPB infestations, and prescribed fire, respectively. The average period between first and last detection of individual juveniles was 10 weeks (range = 2 to 42 weeks).

**Figure 2:**
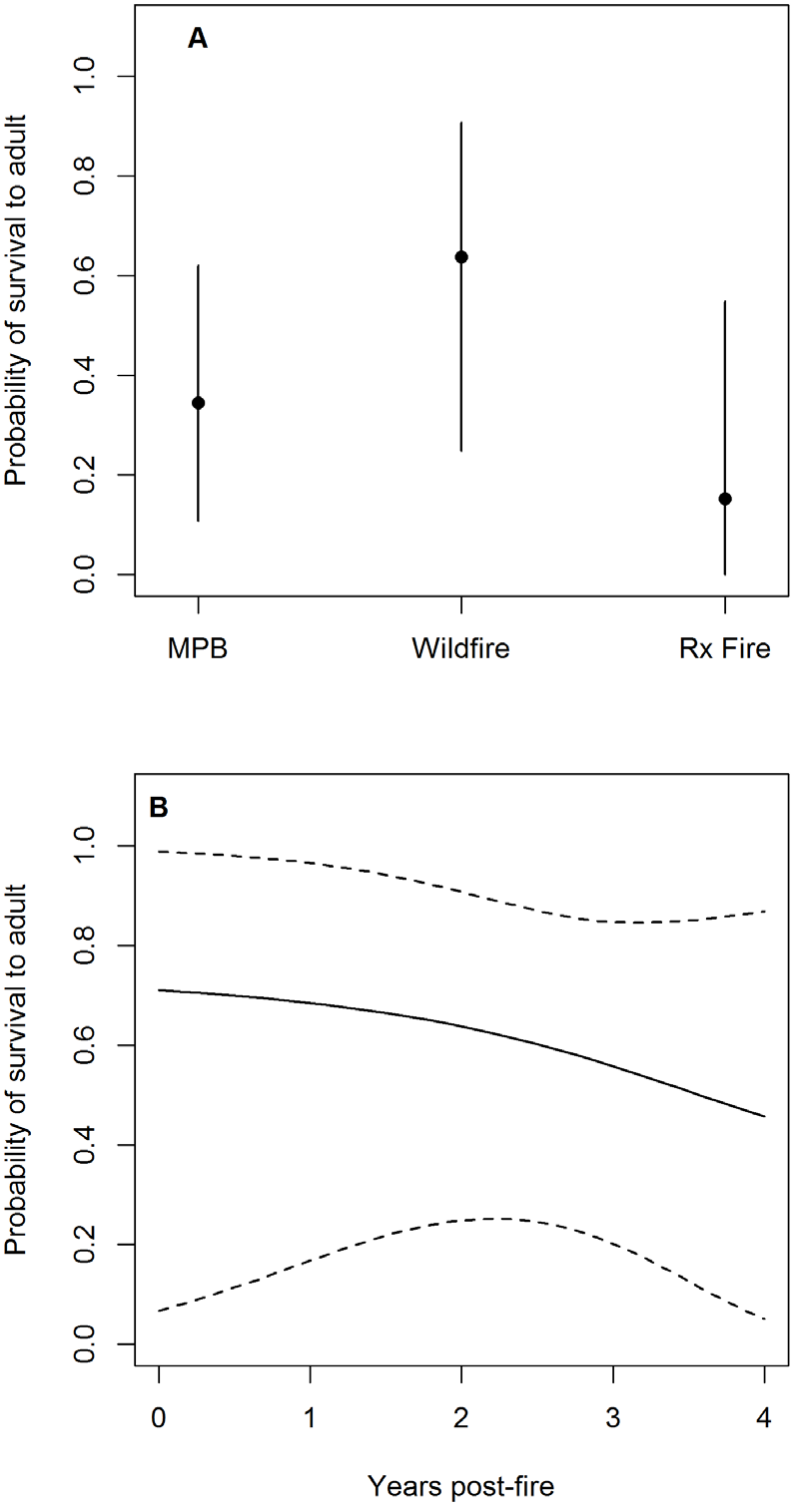
Estimated probabilities of juvenile black-backed woodpeckers surviving to adults in the Black Hills, South Dakota . (a) Mean posterior distribution and 95% CI of the probability juvenile black-backed woodpeckers survive to the adult stage-class in habitat created by wildfire, prescribed (Rx) fire, and mountain pine beetle (MPB) infestations in the Black Hills, SD. Plots of survival probabilities assume habitat created by wildfire and prescribed fire are both 2 years post-fire. (b) Mean posterior distribution and 95% CI of the probability a juvenile black-backed woodpecker survives to the adult stage-class as a function of time since wildfire. This figure assumes a juvenile in habitat created by wildfire, but the trend is similar in habitat created by prescribed fire.

Patterns of habitat-specific juvenile survival probability closely tracked patterns of habitat-specific adult survival probability. The mean probability of juvenile black-backed woodpeckers surviving to the adult stage class (defined as the probability of surviving 21 time steps [42 weeks] post-fledging, Appendix S1) was greatest in habitat created by wildfire, intermediate in habitat created by MPB infestations, lowest in habitat created by prescribed fire, and declined as the time since fire increased (Table S2). For example, the mean probability a juvenile survived to the adult stage class was 0.64 (95% CI = [0.25, 0.91]) in 2-year post-wildfire habitat, 0.35 (95% CI = [0.11, 0.62]) in MPB infestations, and 0.15 (95% CI = [0.00, 0.55]) in 2-year post-prescribed fire habitat (Fig. 2). Mean survival probability was lowest immediately after fledging, and increased as the number of time steps fledged increased (Table S2).

### Nest Success and Number of Young Fledged

We suspected some nest failures were related to capture with the hoop net. We thus censored 14 capture-related nest failures, though we retained detection histories through the last day each nest was observed active. We estimated the probability of a nest successfully fledging at least one young from 95 nests: 40, 35, and 20 in habitat created by wildfire, MPB infestations and prescribed fire, respectively.

**Figure 3:**
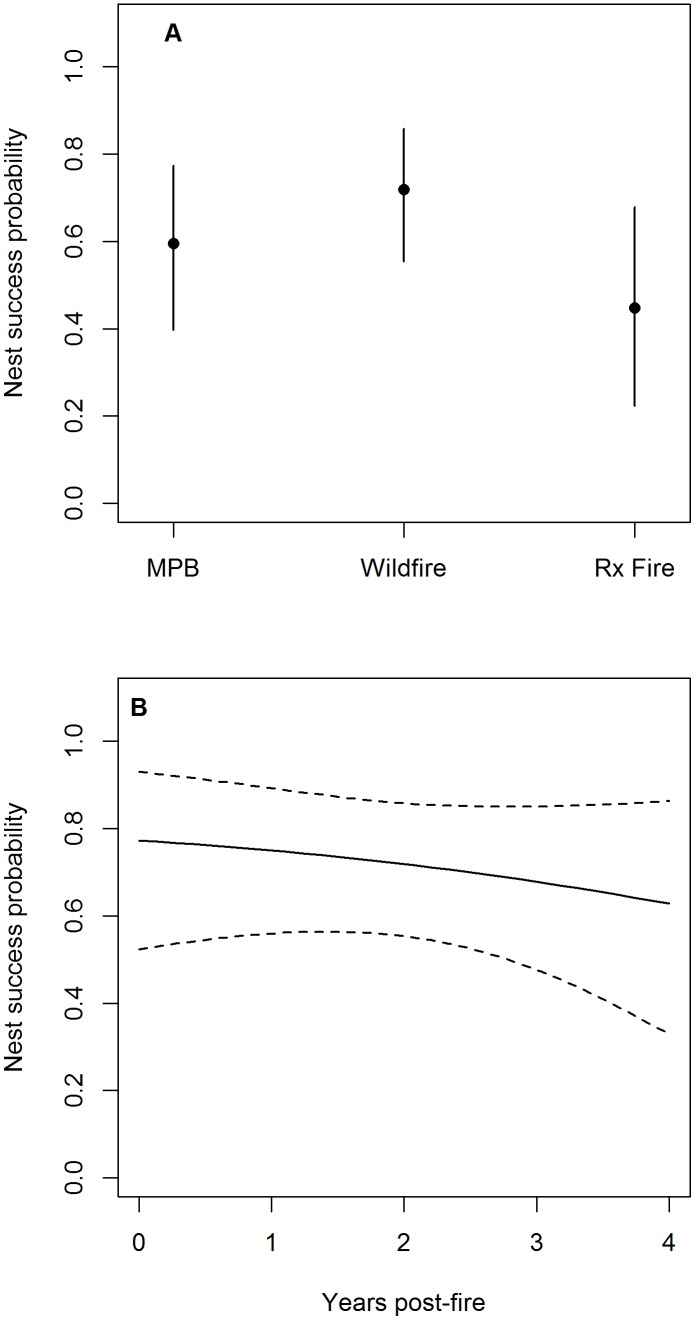
Estimated probabilities of black-backed woodpecker nests successfully fledging at least one young. (a) Mean posterior distribution and 95% credible intervals of the probability a black-backed woodpecker nest successfully fledged at least 1 young in habitat created by wildfire, prescribed (Rx) fire, and mountain pine beetle (MPB) infestations in the Black Hills, SD. This plot assumes nests in habitat created by wildfire and prescribed fire are both 2-years post-fire. (b) Mean posterior distribution and 95% credible intervals of the probability a nest successfully fledges at least 1 young as a function of time since fire. This figure assumes a nest in habitat created by wildfire, but the trend is similar in habitat created by prescribed fire.

Patterns of habitat-specific nest success followed similar patterns as habitat-specific adult and juvenile survival. The mean probability of a nest fledging at least one young was greatest in habitat created by wildfire, intermediate in habitat created by MPB infestations, lowest in habitat created by prescribed fire, and decreased as time since fire increased (Table S3). For example, the mean probability of successfully fledging at least 1 young was 0.72 (95% CI = [0.55, 0.86]) in 2-year post-wildfire habitat, 0.60 (95% CI = [0.40, 0.77]) in MPB infestations, and 0.45 (95% CI = [0.22, 0.68]) in 2-year post-prescribed fire habitat (Fig. 3).

We estimated the expected number of young fledged per successful nest from 50 successful nests: 23, 18, and 9 in habitat created by wildfire, MPB infestations, and prescribed fire, respectively. The expected number of young fledged per successful nest was greatest in habitat created by prescribed fire (mean fledged = 2.05, 95% CI = [1.44, 2.95]) and nearly identical between habitat created by wildfire (mean fledged = 1.80, 95% CI = [1.44, 2.28]) and MPB infestations (mean fledged = 1.81, 95% CI = [1.41, 2.35], Table S4).

### Habitat-specific Population Growth Rates

**Figure 4:**
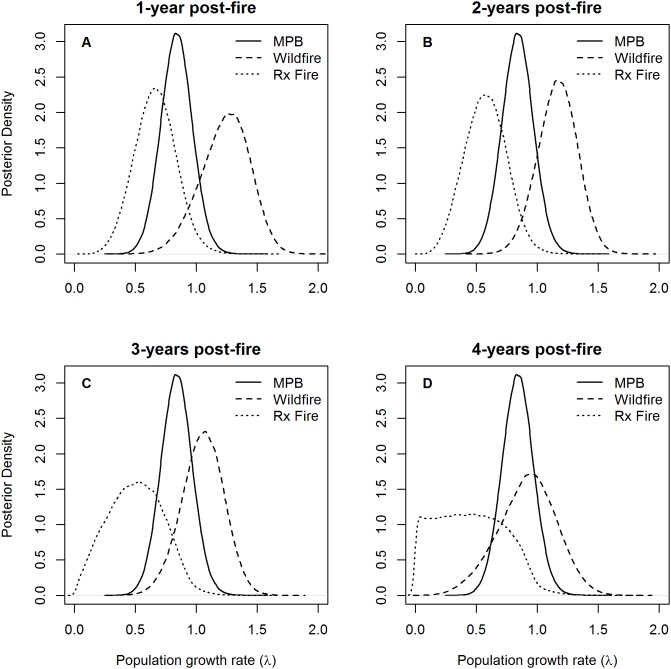
Posterior densities of habitat-specific population growth rates of black-backed woodpeckers in the Black Hills, SD . Plots represent (a) 1-year, (b) 2-years, (c) 3-years, and (d) 4-years post-fire in habitat created by wildfire and prescribed fire (Rx Fire). Note that the posterior density of population growth rates (λ) in mountain pine beetle (MPB) infestations remains constant across panels, since λ in MPB infestations was not estimated as a function of time post-disturbance.

Mean posterior distributions of population growth rates (*λ*) were positive only in habitat created by wildfire. For example, 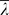 = 1.16 in 2-year post-wildfire habitat and 84% of the posterior density of estimated population growth rate was >1 (Fig. 4b). Mean population growth rates were negative in habitat created by MPB infestations (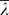 = 0.84) and 11% of the posterior density of estimated population growth rate was >1. Mean population growth rates were also negative in habitat created by prescribed fire. For example, 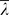 = 0.57 in 2-year post-prescribed fire habitat and <1% of the posterior density of estimated population growth rate was >1. Variation in adult and juvenile survival rates consistently explained the most variation in population growth rates, while reproductive rates explained little variation in population growth rates (Table 2, Fig. S3).

**Table 2:** Proportion of variation in black-backed woodpecker population growth rates explained by variation in component demographic rates.

	Variation Explained (r^2^)^a^		
Demographic Rate	Wildfire	MPB Infestation	Prescribed Fire
Adult Survival	0.34	0.58	0.80
Juvenile Survival	0.48	0.31	0.17
Nest Success	0.08	0.06	0.01
No. Young Fledged	0.09	0.04	0.01

Life-stage simulation analysis was based on 300,000 random draws from the posterior distribution of each demographic rate.

a This table assumes 2-year post wildfire and prescribed fire.

We obtained reasonably distinct posterior distributions of asymptotic growth rates among habitats created by wildfire, prescribed fire, and MPB infestations despite broad and sometimes overlapping posterior distributions of individual demographic rates for two main reasons. First, annual adult survival probability, the probability a juvenile survived to an adult, and the probability of successfully fledging at least 1 young were all identically ranked across habitats. As a consequence, estimated asymptotic growth rates were highest in wildfire and lowest in prescribed fire, despite sometimes considerable uncertainty in component demographic rates. Second, asymptotic growth rates were highly sensitive to both adult and juvenile survival probabilities. As a consequence, small changes in estimated survival probabilities could mean the difference between a growing or a declining population.

## Discussion

This study helps clarify the relative value of wildfire, prescribed fire, and MPB infestations to black-backed woodpeckers in the Black Hills, South Dakota. Mean population growth rates were positive only in habitat created by summer wildfire, while mean population growth rates were negative in habitats created by MPB infestations and fall prescribed fire. These findings support long-standing hypotheses that recently burned forests are population sources for black-backed woodpeckers [10]. Further, the negative population growth we observed in habitat created by fall prescribed fire indicate this management tool, under the conditions we evaluated, is not a viable substitute for summer wildfire.

Habitats created by wildfire and prescribed fire differed at our study sites in two primary ways. First, managers treated sites with prescribed fire in September and October, while wildfire sites burned during June and July. This difference in timing may affect post-fire arthropod communities. For example, some species of wood-boring beetles can detect compounds emitted from burning wood [49] and can rapidly colonize burned forest. These beetles may not be active during autumn months and may be unable to immediately colonize late-season burns (MAR, *personal observation*). Second, the prescribed fire study sites tended to be smaller in area, burn at lower severity, and thus kill fewer trees relative to the wildfire study sites, which may result in different predator communities between the two disturbance types [50].

Our sensitivity analysis provides insight into how these potential differences may affect population growth rates. In all habitats, population growth rates were most sensitive to changes in adult and juvenile survival. If the timing of prescribed fire affects wood-boring beetle abundance, this may affect the food resources available to black-backed woodpeckers, which may in turn impact survival rates. This may particularly affect juvenile survival, since fledglings rely on provisioning from adults for several weeks post-fledging. Survival rates may also be affected by potential differences in predator communities between habitats. For example, Northern Goshawks, a known predator of black-backed woodpeckers in the Black Hills [51], preferentially nest in closed canopy forest [52] and may be less abundant in severely burned forest relative to unburned forest or forest that burned at low severity.

We were surprised that mean population growth rates were negative in habitat created by MPB infestations, since woodpeckers readily occupy and successfully breed in such habitat [17], [18]. However, our finding is consistent with previous hypotheses that black-backed woodpeckers specialize on recently burned forests [30]. Furthermore, black-backed woodpeckers are much more likely to move to burned forest, relative to their availability, than to MPB infestations [51] and home range sizes in recently burned forests are much smaller than home range sizes in MPB infestations [53]. These findings may thus help explain regional differences in the use of MPB infestations. Black-backed woodpeckers are only documented using MPB infestations in isolated portions of their range, where recently burned forest may be relatively rare across the landscape and woodpeckers may face dispersal constraints (e.g., the limited extent of forested habitat in the Black Hills). In contrast, black-backed woodpeckers may rarely use MPB infestations in more contiguous portions of their range if there is a greater relative availability of recently burned forests and woodpeckers do not face such dispersal constraints.

Habitat created by MPB infestations likely harbors some value to black-backed woodpeckers, even if mean population growth rates in this habitat were negative. Indeed, 95% CIs of estimated growth rates overlapped 1, suggesting the potential for positive population growth in this habitat during some years. It is likely that mean population growth rates in MPB infestations are intermediate between early post-wildfire habitat and undisturbed forest, although additional research is needed to understand demographic rates of black-backed woodpeckers in undisturbed forests. Additionally, adult, juvenile, and nest survival all declined as burned forests aged and mean population growth rates in 4-year post-wildfire forests were similar to MPB infestations (Fig. 4d). Mountain pine beetle infestations may thus improve in relative value to black-backed woodpeckers as post-wildfire habitat ages and may help buffer population declines when recently burned forest is not available.

The differences in population growth rates between habitat created by wildfire and MPB infestations likely reflects historic disturbance patterns in the Black Hills. There were 7 discrete MPB outbreaks in the Black Hills during the 20^th^ century, including the current infestation [6]. The extent of individual outbreaks can be larger than the total forest area burned in any given year. However, during most years, MPBs exist at ‘endemic’ levels, defined as <1 tree killed per 0.40 ha per year [6]. In contrast, there is a mean 16 year pre-settlement fire-return interval for ponderosa pine forests in Jewel Cave National Park, South Dakota [33]. Mean fire-return intervals reflect the average time between fires at any given location, though fires probably burned different portions of the Black Hills during most years. These fires likely burned at mixed severity, killing some trees while allowing others to live [54], [55].

We do not wish to completely discard the utility of either prescribed fire or MPB infestations as important disturbance agents for black-backed woodpeckers. Large, severe wildfires are not likely to gain widespread acceptance on public lands, and prescribed fire may be the only way to predictably introduce fire. Prescribed fire is a flexible management tool that can be applied in a variety of ways. For example, managers can vary factors including timing, severity, and extent. Further research is needed to determine the mechanisms leading to negative population growth of black-backed woodpeckers in habitat created by prescribed fire so forests can be treated in an appropriate manner. In particular, further research is needed to understand how the timing of prescribed fire may impact post-fire wood-boring beetle abundance, a primary food resource for black-backed woodpeckers [11].

Although our study evaluates demographic rates of black-backed woodpeckers over a range of disturbed forest conditions, black-backed woodpeckers are also known to use undisturbed forest across their range [19]–[21]. Little is known about demographic rates of black-backed woodpeckers in undisturbed forest conditions, though they are known to occur in very low densities in undisturbed forest in the Black Hills [20] and evidence suggests that black-backed woodpeckers may use undisturbed forests only when recently disturbed forest is not available [22]. Throughout our study, 1–5 year post-fire forest comprised approximately 1.0% and active MPB infestations comprised approximately 3.8% of the forested area, respectively, of the Black Hills [51]. Our dataset was comprised of 138 adult and 37 juvenile radio-marked woodpeckers, including permanent dispersal movements of 18 adult and 7 juvenile woodpeckers up to 60 kilometers away from established study sites [51], and all territories included an element of recently disturbed forest. Our failure to observe any territories without an element of wildfire, prescribed fire, or MPB infestations, particularly among dispersing woodpeckers, indicates undisturbed forests were rarely used and played only a minor role in population dynamics during the duration of our study.

Our study is the first to evaluate the demographic response of black-backed woodpeckers to a range of disturbance conditions. This and other studies are necessary to gain a better understanding of the importance of wildfire and other forest disturbances in maintaining viable black-backed woodpecker populations. Our results indicate this species is dependent on early post-wildfire habitat in the Black Hills, South Dakota, underscoring the importance of ensuring recently burned forest is present across the landscape.

## Supporting Information

Figure S1 **Home ranges of black-backed woodpeckers relative to the 4-Mile wildfire in Custer State Park, SD**. The gray shaded polygon represents the extent of the 4-Mile wildfire, individual points represent coordinates of black-backed woodpecker locations obtained via radio-telemetry, and dashed lines represent 95% probability contours of black-backed woodpecker home ranges estimated using kernel density methods [53]. The 95% home range contours were calculated from the points of the same color. Telemetry locations represented in this figure were collected between April 1 2008 and March 31 2009. The white space outside the burn perimeter represents areas not burned by the 4-Mile wildfire.
(TIF)

Figure S2 **Home ranges of black-backed woodpeckers relative to the East Slate Creek mountain pine beetle infestation**. The green shaded polygons represent <1 year old mountain pine beetle (MPB) infestations as of autumn 2009 [56] and the red shaded polygons represent 1–2 year-old mountain pine beetle infestations as of autumn 2009 [57]. Individual points represent coordinates of black-backed woodpecker locations obtained via radio-telemetry and dashed lines represent 95% probability contours of black-backed woodpecker home ranges estimated using kernel density methods [53]. The 95% home range contours were calculated from the points of the same color. Telemetry locations represented in this figure were collected between April 1 2009 and March 31 2010. The white space outside the shaded polygons represent forests not affected by 0–2 year-old MPB infestations as of autumn 2009.
(TIF)

Figure S3 **Results from life stage simulation analysis evaluating sensitivity of black-backed woodpecker asymptotic population growth rates.** Each point represents an estimate of asymptotic population growth rates (λ) corresponding to a random realization from the posterior distribution of A) annual adult survival probability, B) the probability a juvenile survives to an adult, C) the probability a nest successfully fledges at least 1 young, and D) the expected number of females fledged per successful nest. The coefficient of determiniation (*r* ^2^) is calculated by regressing λ against the corresponding demographic rate [48]. This analysis is based on 300,000 random draws from the posterior distribution of each demographic rate.
(TIFF)

Table S1 **Summary of posterior distributions of parameters included in the adult survival model.**
(PDF)

Table S2 **Summary of posterior distributions of parameters included in the juvenile survival.**
(PDF)

Table S3 **Summary of posterior distributions of parameters included in the nest survival model.**
(PDF)

Table S4 **Summary of posterior distributions of parameters included in the number fledged model.**
(PDF)

Appendix S1 **Statistical methods for modeling demographic rates of black-backed woodpeckers.**
(PDF)
