# Correction: The Role of Wildfire, Prescribed Fire, and Mountain Pine Beetle Infestations on the Population Dynamics of Black-Backed Woodpeckers in the Black Hills, South Dakota

**DOI:** 10.1371/journal.pone.0106390

**Published:** 2014-08-22

**Authors:** The PLOS ONE Staff

In the Materials and Methods section, there are errors in the first paragraph of Estimating Adult and Juvenile Survival. The labels in the first equation should read “state at time *t*-1” and “state at time *t*.” Please see the correct equation here:



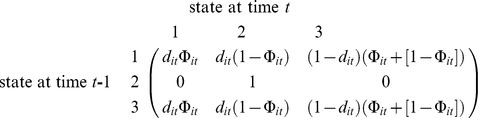



There is an error in the second equation. Please see the correct equation here:



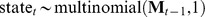



The penultimate sentence of the paragraph should read “Survival probability, denoted Ф*_it_*, is the probability woodpecker *i* is alive at the end of time step *t*.”
