# Supplementary figures and images for: The Role of Wildfire, Prescribed Fire, and Mountain Pine Beetle Infestations on the Population Dynamics of Black-Backed Woodpeckers in the Black Hills, South Dakota
Source: PLoS One. 2014 Apr 15;9(4):e94700. doi: 10.1371/journal.pone.0094700 (PMC3988106; doi:10.1371/journal.pone.0094700)

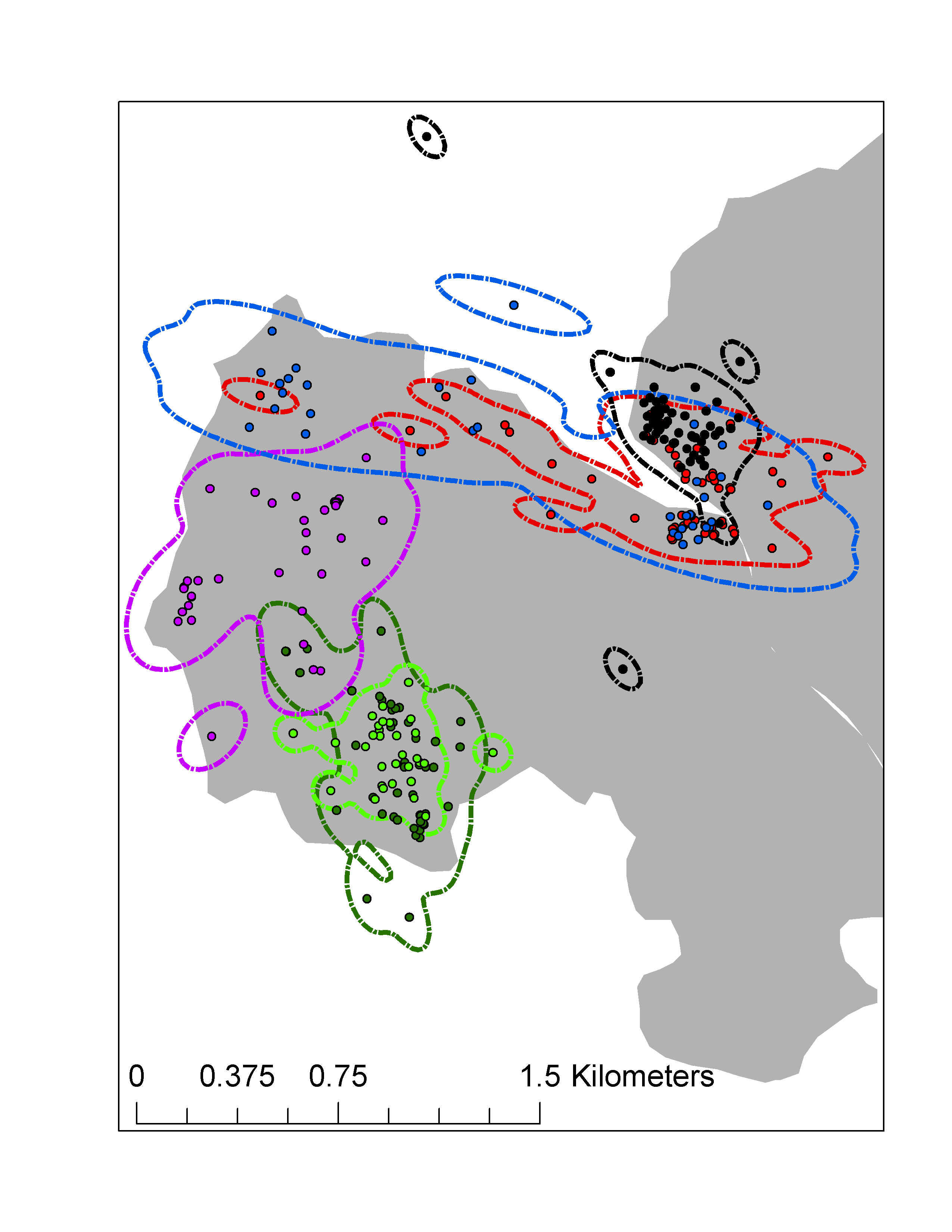

Supplement: Figure S1 — Home ranges of black-backed woodpeckers relative to the 4-Mile wildfire in Custer State Park, SD. The gray shaded polygon represents the extent of the 4-Mile wildfire, individual points represent coordinates of black-backed woodpecker locations obtained via radio-telemetry, and dashed lines represent 95% probability contours of black-backed woodpecker home ranges estimated using kernel density methods [53]. The 95% home range contours were calculated from the points of the same color. Telemetry locations represented in this figure were collected between April 1 2008 and March 31 2009. The white space outside the burn perimeter represents areas not burned by the 4-Mile wildfire. (TIF) [file pone.0094700.s001.tif]

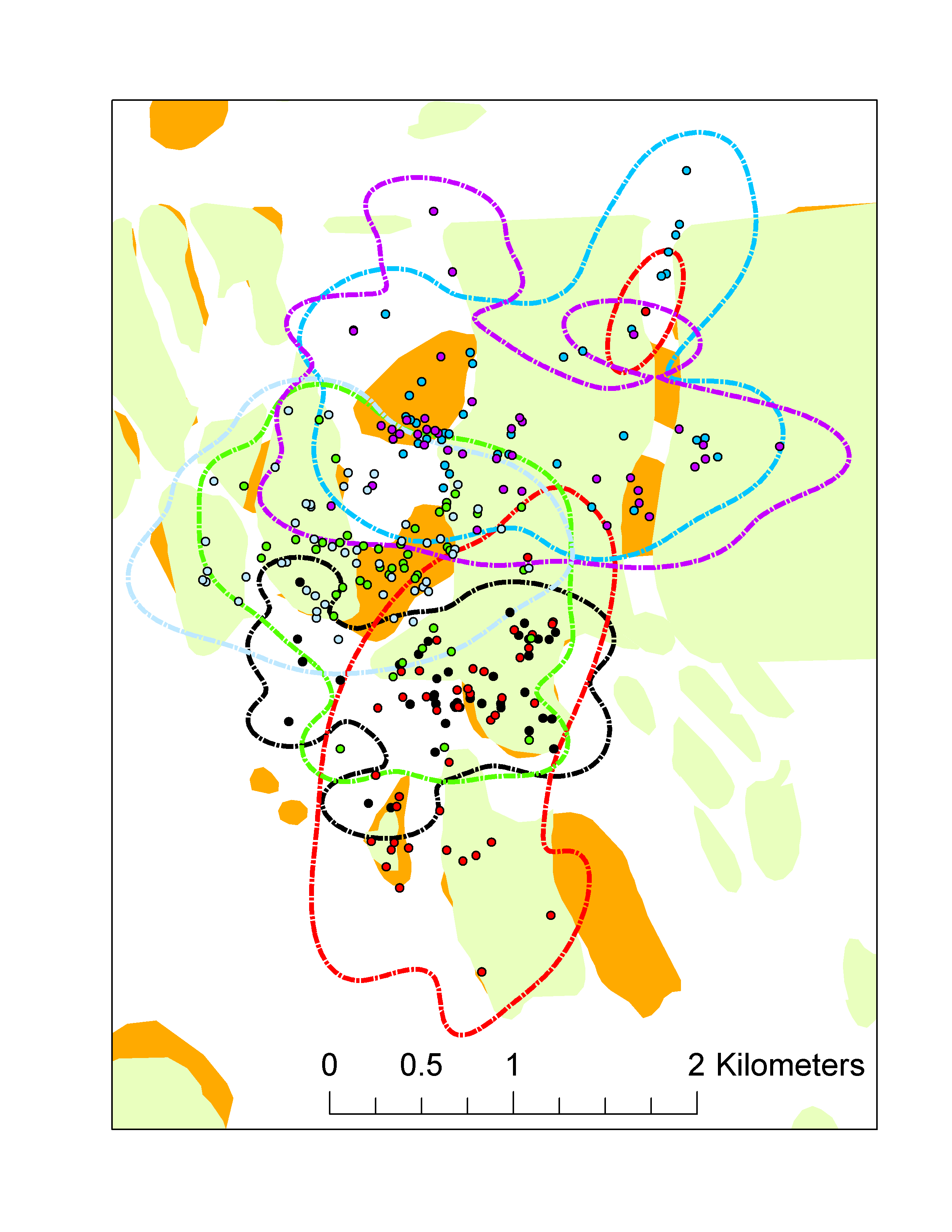

Supplement: Figure S2 — Home ranges of black-backed woodpeckers relative to the East Slate Creek mountain pine beetle infestation. The green shaded polygons represent <1 year old mountain pine beetle (MPB) infestations as of autumn 2009 [56] and the red shaded polygons represent 1–2 year-old mountain pine beetle infestations as of autumn 2009 [57]. Individual points represent coordinates of black-backed woodpecker locations obtained via radio-telemetry and dashed lines represent 95% probability contours of black-backed woodpecker home ranges estimated using kernel density methods [53]. The 95% home range contours were calculated from the points of the same color. Telemetry locations represented in this figure were collected between April 1 2009 and March 31 2010. The white space outside the shaded polygons represent forests not affected by 0–2 year-old MPB infestations as of autumn 2009. (TIF) [file pone.0094700.s002.tif]

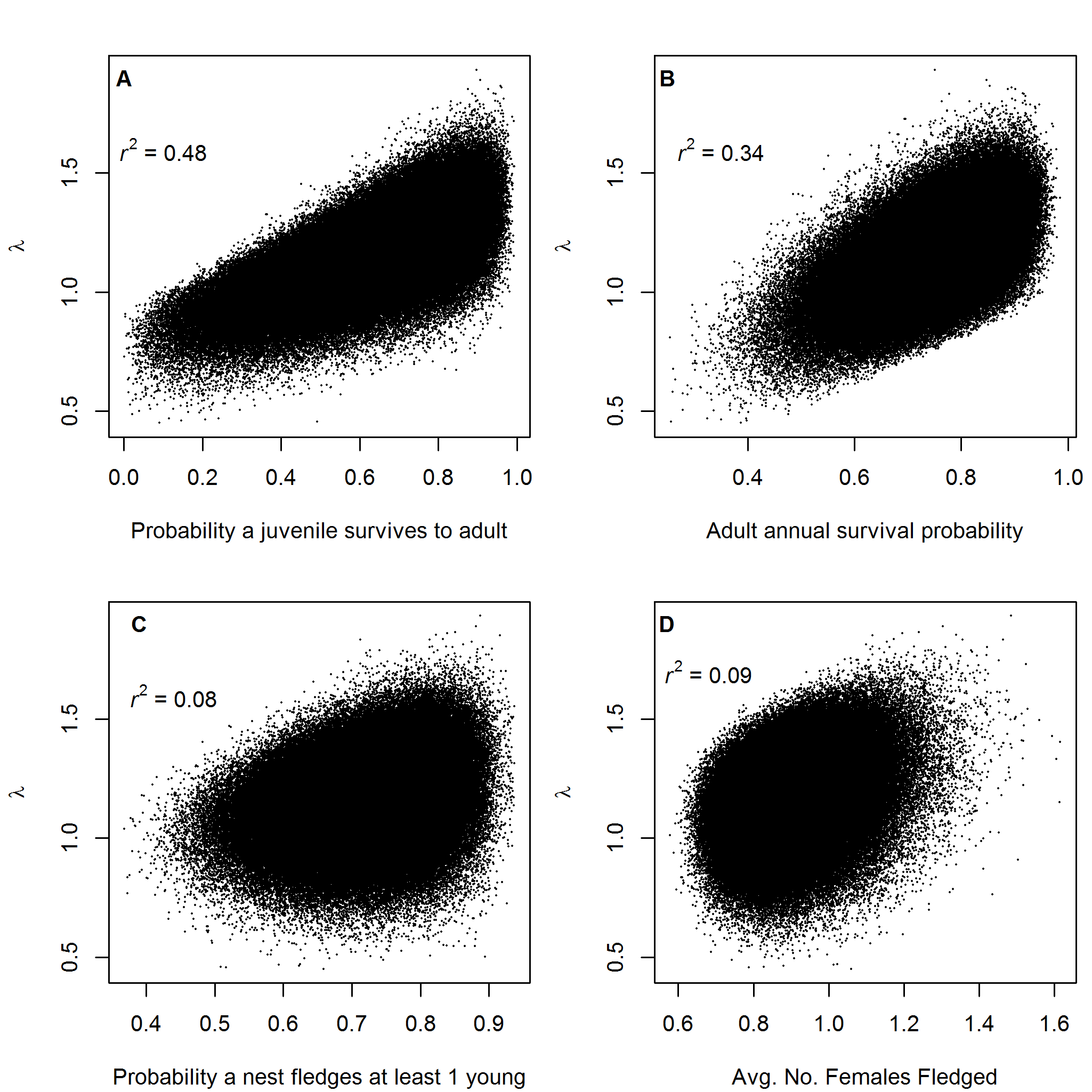

Supplement: Figure S3 — Results from life stage simulation analysis evaluating sensitivity of black-backed woodpecker asymptotic population growth rates. Each point represents an estimate of asymptotic population growth rates (λ) corresponding to a random realization from the posterior distribution of A) annual adult survival probability, B) the probability a juvenile survives to an adult, C) the probability a nest successfully fledges at least 1 young, and D) the expected number of females fledged per successful nest. The coefficient of determiniation (r 2) is calculated by regressing λ against the corresponding demographic rate [48]. This analysis is based on 300,000 random draws from the posterior distribution of each demographic rate. (TIFF) [file pone.0094700.s003.tif]
