# Supplementary material for: The Role of Wildfire, Prescribed Fire, and Mountain Pine Beetle Infestations on the Population Dynamics of Black-Backed Woodpeckers in the Black Hills, South Dakota
Source: PLoS One. 2014 Apr 15;9(4):e94700. doi: 10.1371/journal.pone.0094700 (PMC3988106; doi:10.1371/journal.pone.0094700)
Supplement: Table S1 — Summary of posterior distributions of parameters included in the adult survival model. (PDF) [file pone.0094700.s004.pdf]

Parameter	Median	Variance	Lower 95% CI	Upper 95% CI
Survival				
β_0^{ad} (intercept)	3.202	0.184	2.426	4.106
β_1^{ad} (male)	0.056	0.168	-0.755	0.855
β_2^{ad} (breeding)	0.352	0.172	-0.474	1.162
β_3^{ad} (wildfire)	0.728	0.807	-0.902	2.627
β_4^{ad} (rx fire)	-0.185	0.503	-1.533	1.255
β_5^{ad} (year)	-0.160	0.120	-0.837	0.523
Detection Probability				
α_0 (intercept)	-3.067	0.012	-3.287	-2.859
α_1 (transmitter)	4.884	0.022	4.599	5.180
