# Supplementary material for: The Role of Wildfire, Prescribed Fire, and Mountain Pine Beetle Infestations on the Population Dynamics of Black-Backed Woodpeckers in the Black Hills, South Dakota
Source: PLoS One. 2014 Apr 15;9(4):e94700. doi: 10.1371/journal.pone.0094700 (PMC3988106; doi:10.1371/journal.pone.0094700)
Supplement: Table S2 — Summary of posterior distributions of parameters included in the juvenile survival. (PDF) [file pone.0094700.s005.pdf]

Parameter	Median	Variance	Lower 95% CI	Upper 95% CI
Survival				
β_0^{juv} (intercept)	1.599	0.248	0.696	2.650
β_1^{juv} (wildfire)	1.579	2.054	-0.990	4.634
β_2^{juv} (rx fire)	-0.170	0.700	-1.764	1.524
β_3^{juv} (year)	-0.315	0.215	-1.233	0.587
β_4^{juv} (time steps fledged)	0.719	0.111	0.093	1.405
Detection Probability				
α_0 (intercept)	-2.089	0.017	-2.355	-1.842
α_1 (transmitter)	3.152	0.041	2.764	3.556
