# Supplementary material for: The Role of Wildfire, Prescribed Fire, and Mountain Pine Beetle Infestations on the Population Dynamics of Black-Backed Woodpeckers in the Black Hills, South Dakota
Source: PLoS One. 2014 Apr 15;9(4):e94700. doi: 10.1371/journal.pone.0094700 (PMC3988106; doi:10.1371/journal.pone.0094700)
Supplement: Table S3 — Summary of posterior distributions of parameters included in the nest survival model. (PDF) [file pone.0094700.s006.pdf]

Parameter	Median	Variance	Lower 95% CI	Upper 95% CI
θ_0 (intercept)	4.242	0.111	3.651	4.939
θ_1 (wildfire)	0.768	0.394	-0.410	2.016
θ_2 (rx fire)	-0.167	0.396	-1.292	1.197
θ_3 (year)	-0.151	0.043	-0.576	0.240
