# Supplementary material for: The Role of Wildfire, Prescribed Fire, and Mountain Pine Beetle Infestations on the Population Dynamics of Black-Backed Woodpeckers in the Black Hills, South Dakota
Source: PLoS One. 2014 Apr 15;9(4):e94700. doi: 10.1371/journal.pone.0094700 (PMC3988106; doi:10.1371/journal.pone.0094700)
Supplement: Table S4 — Summary of posterior distributions of parameters included in the number fledged model. (PDF) [file pone.0094700.s007.pdf]

Parameter	Median	Variance	Lower 95% CI	Upper 95% CI
π_0 (intercept)	0.258	0.069	-0.312	0.719
π_1 (wildfire)	-0.002	0.125	-0.687	0.701
π_2 (rx fire)	0.210	0.174	-0.639	1.003
