# Supplementary material for: The Role of Wildfire, Prescribed Fire, and Mountain Pine Beetle Infestations on the Population Dynamics of Black-Backed Woodpeckers in the Black Hills, South Dakota
Source: PLoS One. 2014 Apr 15;9(4):e94700. doi: 10.1371/journal.pone.0094700 (PMC3988106; doi:10.1371/journal.pone.0094700)
Supplement: Appendix S1 — Statistical methods for modeling demographic rates of black-backed woodpeckers. (PDF) [file pone.0094700.s008.pdf]

Appendix S1: Statistical Methods for Modeling Demographic Rates of Black-backed Woodpeckers

Detection Probability

We modeled detection probability as a function of whether woodpecker *i* had an active transmitter during timestep *t*:

$$d_{it} = \text{logit}^{-1}(\alpha_0 + \alpha_1 \tau_{it})$$

where $\tau_{it} = 1$ if woodpecker *i* has an active transmitter during timestep *t*, 0 otherwise, and α_0 and α_1 are regression coefficients. We assumed normal ($\mu = 0$, $\sigma^2 = 1{,}000$) prior distributions on the parameters α_0 and α_1. Because we do not know exactly how long transmitters lasted if we failed to observe woodpeckers, we assumed adult transmitters were active for 5 time steps (5 months) and juvenile transmitters were active for 7 time steps (14 weeks), unless we observed otherwise (e.g., observed a woodpecker with an inactive transmitter prior to the assumed fail time or observed a woodpecker with an active transmitter beyond the assumed fail time). If transmitters were active beyond the assumed fail time, we assumed the transmitter failed the time step immediately following the last observation with an active transmitter.

Adult Survival Probability

We modeled monthly adult survival probability as a function of sex, season, habitat, and number of years post-fire:

$$\varphi_{it}^{\text{ad}} = \text{logit}^{-1}\left(\beta_0^{\text{ad}} + \beta_1^{\text{ad}}\varsigma_i + \beta_2^{\text{ad}}\gamma_{it} + \beta_3^{\text{ad}}\omega_{it}^{\text{ad}} + \beta_4^{\text{ad}}x_{it}^{\text{ad}} + \beta_5^{\text{ad}}\eta_{it}^{\text{ad}}\left[\omega_{it}^{\text{ad}} + x_{it}^{\text{ad}}\right]\right) \qquad \text{(Eqn. 1)}$$

where $\varsigma_i = 1$ if adult woodpecker *i* is male, 0 if female, $\gamma_{it} = 1$ if the observation of adult woodpecker *i* is made during the breeding season (April –September), 0 if made during the non-breeding season, $\omega_{it}^{\text{ad}} = 1$ if adult woodpecker *i* is occupying habitat created by wildfire at the

end of time step t, 0 otherwise, x_{it}^{ad} = 1 if adult woodpecker i is occupying habitat created by prescribed fire at the end of time step t, 0 otherwise, η_{it}^{ad} is the age (in years) of habitat created by wildfire or prescribed fire adult woodpecker i occupied at the end of time step t $\left(\eta_{it}^{\text{ad}} \in [0,1,2,3,4,5]\right)$, and $\beta_0^{\text{ad}},...,\beta_5^{\text{ad}}$ are regression coefficients. We assumed normal (μ = 0, σ^2 = 1,000) prior distributions on the parameters $\beta_0^{\text{ad}},...,\beta_5^{\text{ad}}$. Note that the time since fire term applies only to woodpeckers occupying habitat created by wildfire or prescribed fire, since $\omega_{it}^{\text{ad}} + x_{it}^{\text{ad}} = 0$ if adult woodpecker i is occupying habitat created by MPB infestations at the end of time step t.

Juvenile Survival Probability

We modeled juvenile bi-weekly survival probability as a function of habitat (wildfire, MPB, and prescribed fire), the number of years post-fire, and the number of two-week time intervals since fledging:

$$\varphi_{it}^{\text{juv}} = \text{logit}^{-1}\left(\beta_0^{\text{juv}} + \beta_1^{\text{juv}}\omega_{it}^{\text{juv}} + \beta_2^{\text{juv}}x_{it}^{\text{juv}} + \beta_3^{\text{juv}}\eta_{it}^{\text{juv}}\left[\omega_{it}^{\text{juv}} + x_{it}^{\text{juv}}\right] + \beta_4^{\text{juv}}\log\left[\delta_{it}\right]\right) \qquad \text{(Eqn. 2)}$$

where ω_{it}^{juv} = 1 if juvenile i occupied habitat created by wildfire at the end of time step t, 0 otherwise, x_{it}^{juv} = 1 if juvenile i occupied habitat created by prescribed fire at the end of time step t, 0 otherwise, η_{it}^{juv} is the age (in years) of habitat created by wildfire or prescribed fire juvenile i occupied at the end of time step t $\left(\eta_{it}^{\text{juv}} \in [0,1,2,3,4,5]\right)$, δ_{it} is the number of time steps juvenile i has been fledged from the nest at the end of time step t $\left(\delta_{it} \in [1,2,...,21]\right)$, and $\beta_0^{\text{juv}},...,\beta_4^{\text{juv}}$ are regression coefficients. Modeling juvenile survival probability as a function of the $\log_e$ number of time steps fledged allowed survival probability to change in a non-linear manner. We assumed normal (μ = 0, σ^2 = 1,000) prior distributions on the parameters $\beta_0^{\text{juv}},...,\beta_4^{\text{juv}}$.

We fit adult and juvenile MSMR models in WinBUGS [1] via the R2WinBUGS interface [2]. We simulated posterior distributions of all regression coefficients from 3 Markov chains, each of which ran for 5.5 million iterations and discarding the first 500,000 iterations as burn-in. There was evidence of correlation within Markov chains so we kept every 50th iteration to reduce correlation between successive draws. Estimates of posterior distributions are thus based on 100,000 draws from each Markov chain for a total of 300,000 random draws from the posterior distribution of each regression coefficient. The Brooks-Gelman-Rubin convergence diagnostic [3] indicated adequate convergence $\left(\hat{R}=1\right)$ for all regression coefficients.

Daily Nest Survival Probability

We assumed survival (y_{it} = 1) or failure (y_{it} = 0) of nest *i* during day *t* was a Bernoulli random variable:

$$y_{it} \sim \text{Bernoulli}(\psi_{it})$$

where ψ_{it} is the probability nest *i* survives day *t*. We further modeled daily survival probability as a function of habitat and time since fire:

$$\psi_{it} = \text{logit}^{-1}\left(\theta_0 + \theta_1\omega_i^{\text{nest}} + \theta_2 x_i^{\text{nest}} + \theta_3\eta_i^{\text{nest}}\left[\omega_i^{\text{nest}} + x_i^{\text{nest}}\right]\right) \qquad \text{(Eqn. 3)}$$

where ω_i^{nest} = 1 if nest *i* is located in habitat created by wildfire, 0 otherwise, x_i^{nest} = 1 if nest *i* is located in habitat created by prescribed fire, 0 otherwise, η_i^{nest} is the age (in years) of habitat created by wildfire or prescribed where nest *i* was located $\left(\eta_i^{\text{nest}} \in \left[0,1,2,3,4,5\right]\right)$, and $\theta_0, \ldots, \theta_3$ are regression coefficients. We assumed normal ($\mu = 0$, $\sigma^2 = 1,000$) prior distributions on the parameters $\theta_0, \ldots, \theta_3$.

We fit nest survival models in WinBUGS via the R2WinBUGS interface. We simulated posterior distributions of all regression coefficients from 3 Markov chains, each of which ran for 550,000 iterations, with the first 50,000 iterations discarded as burn-in. There was evidence of correlation within Markov chains so we kept every 5th iteration to reduce correlation between successive draws. Estimates of posterior distributions are thus based on 100,000 draws from each Markov chain for a total of 300,000 random draws from the posterior distribution of each regression coefficient. The Brooks-Gelman-Rubin convergence diagnostic indicated adequate convergence $\left(\hat{R}=1\right)$ for all regression coefficients.

Expected Number of Young Fledged

We modeled the number of young fledged (z_i) from successful nest i using a zero-truncated Poisson model:

$$z_i \sim \text{truncated Poisson}(\rho_i)$$

where $\mathrm{k}_i = \rho_i / \left(1 - e^{-\rho_i}\right)$ is the expected number of young fledged from nest i [4]. We modeled the number of young fledged as a function of habitat:

$$\rho_i = \exp\left(\pi_0 + \pi_1 \omega_i^{\text{nest}} + \pi_2 x_i^{\text{nest}}\right) \qquad \text{(Eqn. 4)}$$

where π_0, π_1, and π_2 are regression coefficients. We assumed normal ($\mu = 0$, $\sigma^2 = 1{,}000$) prior distributions on the parameters π_0, π_1, and π_2.

A zero-truncated Poisson distribution is not among the distributions offered in the WinBUGS package. We therefore wrote a Metropolis-Hastings algorithm to sample from the posterior distributions of model parameters. We tuned the Metropolis-Hastings algorithm to have an acceptance probability of approximately 20% [5]. We simulated posterior distributions of all regression coefficients from 3 Markov chains, each of which ran for 5.1 million iterations with the first 100,000 iterations discarded as burn-in. To minimize correlation within chains due

to the accept / reject step in the Metropolis-Hastings algorithm, we kept every 50th iteration. Estimates of posterior distributions are thus based on 100,000 draws from each Markov chain for a total of 300,000 random draws from the posterior distribution of each regression coefficient.

Scaling Demographic Rates

We calculated habitat specific fecundity (m_h) as a function of habitat specific nest success and number of young fledged per successful nest:

$$m_h = \varepsilon_h(\kappa_h / 2)$$

where ε_h is habitat specific nest success, defined as the probability a nest in habitat h successfully fledges young. We divided the expected number of young fledged per nest, κ_h, by 2 because we assume a 50:50 fledgling sex ratio. We estimated habitat-specific growth rates by calculating the dominant eigenvalue of projection matrix $\mathbf{A}_h$.

We calculated habitat specific fecundity, annual adult survival probability, and the probability a juvenile survives to the adult stage class as a function of the regression coefficients estimated from demographic analyses above and habitat-specific covariates. We calculated habitat-specific annual female adult survival as:

$$P_h^{\text{ad}} = \text{logit}^{-1}\left(\hat{\beta}_0^{\text{ad}} + \hat{\beta}_2^{\text{ad}}\gamma + \hat{\beta}_3^{\text{ad}} I_{\text{wild}} + \hat{\beta}_4^{\text{ad}} I_{\text{Rx}} + \hat{\beta}_5^{\text{ad}}\eta\left[I_{\text{wild}} + I_{\text{Rx}}\right]\right)^6 \times \text{logit}^{-1}\left(\hat{\beta}_0^{\text{ad}} + \hat{\beta}_3^{\text{ad}} I_{\text{wild}} + \hat{\beta}_4^{\text{ad}} I_{\text{Rx}} + \hat{\beta}_5^{\text{ad}}\eta\left[I_{\text{wild}} + I_{\text{Rx}}\right]\right)^6$$

where I_{wild} and I_{Rx} are indicator variables = 1 if habitat h is wildfire or prescribed fire, respectively, 0 otherwise, and $\hat{\beta}_0^{\text{ad}},...,\hat{\beta}_5^{\text{ad}}$ represent random samples from the posterior distributions of regression coefficients defined in Eqn. 1. Equations are raised to the 6th power because adult survival probability was modeled on monthly time steps. Note the term $\hat{\beta}_2^{\text{ad}}$ represents the regression coefficient for season-specific adult survival probability, which is why

the term is excluded from the equation for 6 months. We calculated the habitat-specific probability a juvenile will survive to an adult as:

$$P_h^{\text{juv}} = \prod_{j=1}^{21} \text{logit}^{-1}\left(\hat{\beta}_0^{\text{juv}} + \hat{\beta}_1^{\text{juv}} I_{\text{wild}} + \hat{\beta}_2^{\text{juv}} I_{\text{Rx}} + \hat{\beta}_3^{\text{juv}} \eta\left[I_{\text{wild}} + I_{\text{Rx}}\right] + \hat{\beta}_4^{\text{juv}} \log\left[j\right]\right)$$

where $\hat{\beta}_0^{\text{juv}},...,\hat{\beta}_4^{\text{juv}}$ represent random samples from the posterior distributions of regression coefficients defined in Eqn. 2. Note that the estimate of the probability a juvenile survives to the adult stage class incorporates a different survival probability for each time step fledged. By multiplying this term over 21 time steps, we assume a fledge date of approximately 24 June and that juveniles transition to the adult stage by approximately 1 April the following year. We calculate habitat-specific nest success as:

$$\varepsilon_h = \text{logit}^{-1}\left(\hat{\theta}_0 + \hat{\theta}_1 I_{\text{wild}} + \hat{\theta}_2 I_{\text{Rx}} + \hat{\theta}_3 \eta\left[I_{\text{wild}} + I_{\text{Rx}}\right]\right)^{36}$$

where $\hat{\theta}_0,...,\hat{\theta}_3$ represent random samples from the posterior distributions of regression coefficients defined in Eqn. 3. Our calculation of habitat-specific nest success assumes a 36 day nesting period (incubation period = 13 days, nestling period = 23 days), which was based on observed length of nesting periods and closely corresponds with the published literature [6], [7]. Our nesting period was three days longer than [6] because we assumed the nest was at risk of failure the day the first egg was laid. Finally, we calculated the expected number of young fledged in habitat *h as*:

$$\text{k}_h = \frac{\rho_h}{1-e^{-\rho_h}},$$

where

$$\rho_h = \exp\left(\hat{\pi}_0 + \hat{\pi}_1 I_{\text{wild}} + \hat{\pi}_2 I_{\text{Rx}}\right)$$

and $\hat{\pi}_0, ..., \hat{\pi}_2$ represent random samples from the posterior distributions of regression coefficients defined in Eqn. 4.

We estimated the posterior distribution of habitat specific fecundity, annual adult survival probability, and the probability a juvenile survives to the adult stage class by repeatedly sampling from the posterior distributions of component regression coefficients. This repeated sampling allowed us to propagate uncertainty in estimated regression coefficients. We repeated this process 300,000 times for each habitat-specific demographic rate. Finally, we estimated the posterior distribution of habitat-specific growth rates by constructing 300,000 random projection matrices for each habitat, with each cell element comprising a random draw from the posterior distribution of habitat-specific adult survival, juvenile survival, and fecundity.

Life-stage Simulation Analysis

We calculated the proportion of variation in population growth rates explained by variation in component demographic rates by first regressing estimates of population growth rates (the dominant eigenvalue of $\mathbf{A}_h$) against the random values of adult survival, juvenile survival, nest success, and expected number of female young fledged used to construct $\mathbf{A}_h$ (life-stage simulation analysis [8]). We next calculated the coefficient of determination (r^2) from each regression model to calculate the proportion of variation in λ explained by variation in each component demographic rate (Fig. S3).

References

1. Gilks WR, Thomas A, Spiegelhalter DJ (1994). A language and program for complex Bayesian modelling. J R Stat Soc Ser D Statistician 43: 169–177.
2. Sturtz S, Ligges U, Gelman A (2005) R2WinBUGS : A package for running WinBUGS. J Stat Softw 12: 1–16.
3. Brooks SP, Gelman A (1998) General methods for monitoring convergence of iterative simulations. J Comput Graph Stat 7: 434–455.
4. David FN, Johnson NL (1952) The truncated Poisson. Biometrics 8: 275–285.
5. King R, Morgan BJT, Gimenez O, Brooks SP (2010) Markov chain monte carlo. In: King R, Morgan BJT, Gimenez O, Brooks SP. Bayesian analysis for Population Ecology. Boca Raton: Taylor & Francis Group. pp. 99-146.
6. Bonnot TW, Rumble MA, Millspaugh JJ (2008) Nest success of black-backed woodpeckers in forests with mountain pine beetle outbreaks in the Black Hills, South Dakota. Condor 110: 450–457.
7. Dixon RD, Saab VA (2000) Black-backed Wooodpecker (Picoides arcticus). In Poole A, editor. The Birds of North America Online. Ithaca: Cornell Lab of Ornithology. Available: http://bna.birds.cornell.edu/bna/species/509. Accessed May 2013.
8. Wisdom MJ, Mills LS, Doak DF (2000) Life stage simulation analysis: Estimating vital-rate effects on population growth for conservation. Ecology 81: 628–641.
